# A translational consideration of intercellular adhesion molecule-1 biology in the perioperative setting

**Published:** 2016

**Authors:** Emily Greenwald, Koichi Yuki

**Affiliations:** 1 Department of Anesthesiology, Perioperative and Pain Medicine, Boston Children’s Hospital; Brandeis University; 2 Department of Anesthesiology, Perioperative and Pain Medicine, Boston Children’s Hospital

**Keywords:** Intercellular adhesion molecule, leukocyte, recruitment

## Abstract

Intercellular adhesion molecule-1 (ICAM-1) is a critical adhesion molecule involved in leukocyte recruitment. Since its discovery in 1986, a large number of studies have been performed to elucidate its role *in vitro* and *in vivo*. Here, we review its role in leukocyte recruitment and consider future steps to take that will enhance our understanding of ICAM-1 biology and its translational application in the perioperative setting.

## Introduction

Our body has multi-layered defense mechanisms against the outside world. Skin, for example, is the most exposed organ of the human body and acts as the first line of defense against foreign organisms present in the environment. Mucosa also acts as a strong barrier. Surgical procedures often disrupt these barriers and introduce various organisms to otherwise sterile sites. Adequate immune function to eradicate these organisms is critical for minimizing perioperative complications.

Leukocytes are major constituents in the immune system and are recruited to contaminated sites to fight against a variety of organisms that may otherwise contribute to increased morbidities and mortalities in the perioperative period. Tissue injury as a result of surgery also triggers acute inflammation to which leukocytes respond, known as ‘sterile inflammation’. The mechanism by which these leukocytes migrate to the site of infection or injury is complex and involves multiple molecules. A sophisticated interaction between circulating leukocytes and the endothelium is an essential component of this response. [1] Intercellular adhesion molecule-1 (ICAM-1) is considered as one of the most critical molecules in leukocyte-endothelium interaction. Here, we review ICAM-1 biology in the context of leukocyte function and discuss its translational application in the perioperative setting.

## The discovery of ICAM-1 and its various forms

**Figure 1:**
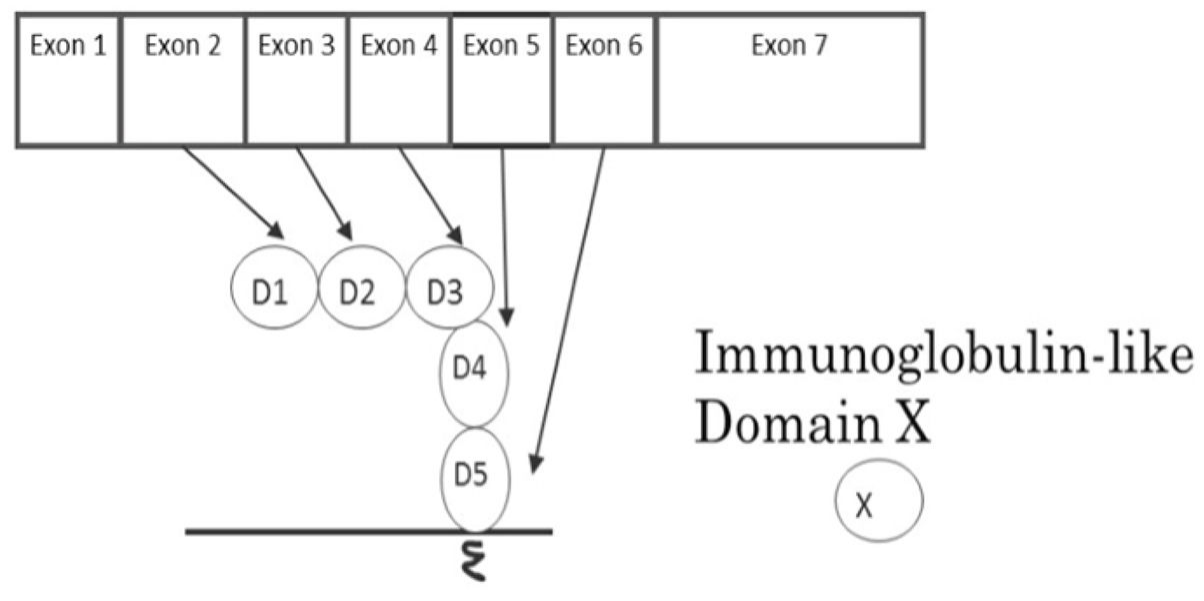
The relationship between ICAM-1 exons and immunoglobulin-like domains.

ICAM-1 was discovered in 1986 as a binding partner for the adhesion molecule leukocyte function-associated antigen-1 (LFA-1). [2] ICAM-1 is encoded by seven exons; the coding region for the human ICAM-1 gene spans 1599 base pairs, while the mouse gene spans 1614 base pairs. The homology between human and mouse ICAM-1 nucleotide sequences is 65% [* homology matching was performed using Gene-tyx-Mac software]. The amino acid sequence homology is 55%, [3] suggesting a high similarity. ICAM-1 is a member of the immunoglobulin supergene family and consists of five homologous immunoglobulin-like domains (D1-D2-D3-D4-D5), one transmembrane domain, and a short cytoplasmic tail. Each immunoglobulin-like domain is encoded by an exon of the ICAM-1 gene, beginning with exon 2. Exon 2 encodes domain 1 (D1), and exon (X) encodes domain (X-1) until exon 6 that encodes domain 5 (D5) (Figure 1). Normally, endothelial cells, monocytes and lymphocytes have low ICAM-1 expression, but pro-inflammatory stimuli such as TNF-α, IL-1 and IFN-γ can upregulate its expression level. [3] Thus, ICAM-1 can be categorized as a pro-inflammatory molecule. Surgical procedures often induce pro-inflammatory responses and likely upregulate ICAM-1 expression on the endothelium.

**Figure 2:**
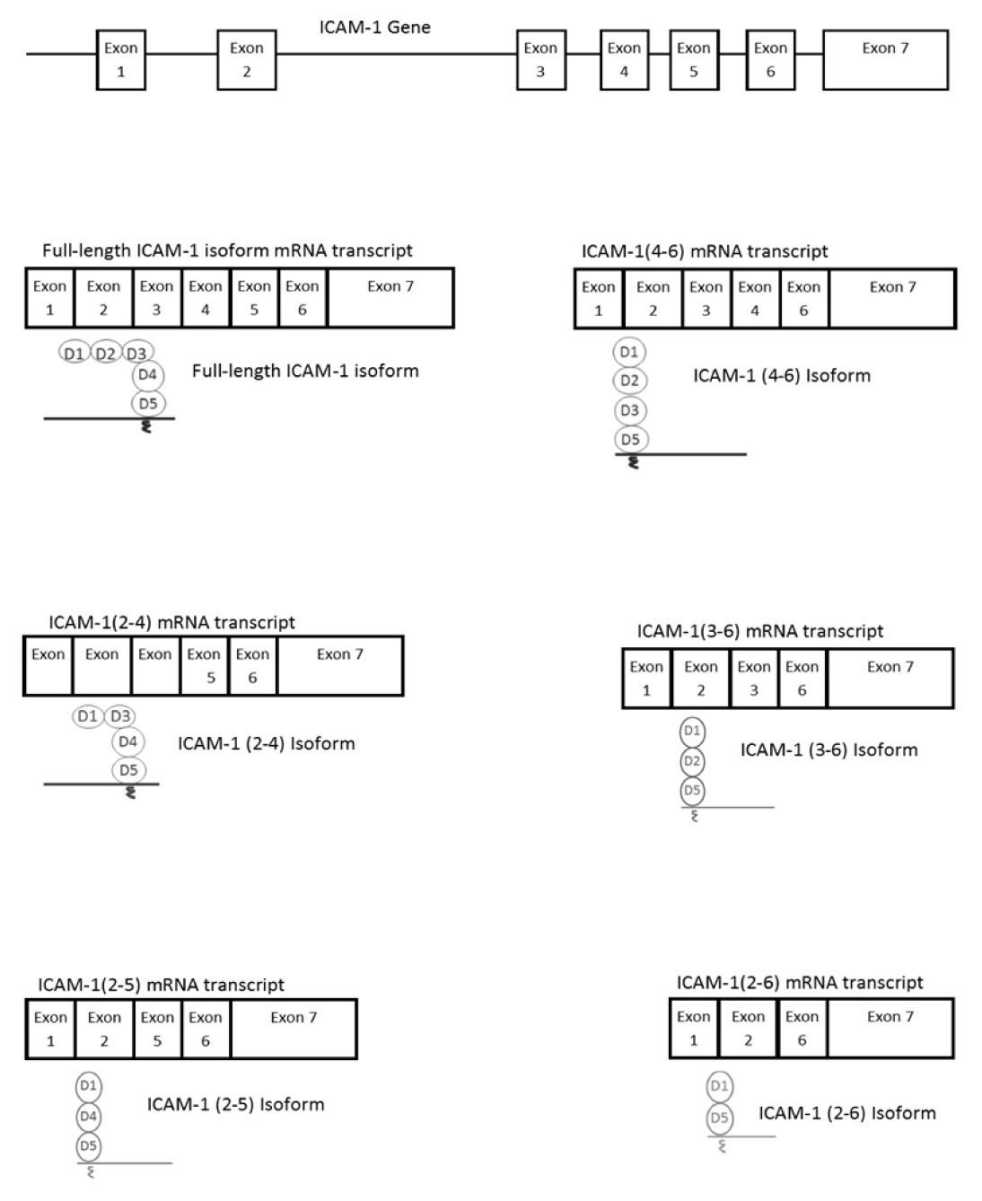
ICAM-1 isoforms

ICAM-1 undergoes alternative splicing and post-translational modifications, and therefore exists in various forms. In mice, ICAM-1 is primarily expressed in the full-length form encoded by all of the exons and contains D1D2D3D4D5, but alternatively spliced isoforms that encode only a part of the immunoglobulin-like domains are also produced. [4] These iso-forms are named D1D2D3D5, D1D3D4D5, D1D2D5, D1D4D5 and D1D5, based on the domains that they contain (Figure 2). These forms of ICAM-1 are membrane-bound. In humans, conclusive evidence of ICAM-1 isoforms has not yet been reported. However, Dustin and collaborators reported two discrete bands specific to ICAM-1 in their study, which may suggest the presence of ICAM-1 isoforms in humans. [5] Both human and mouse ICAM-1 undergo glycosylation. It is well known that ICAM-1 is expressed on the cell surface as the membrane-bound form, and proteolytic cleavage of membrane-bound ICAM-1 (mICAM-1) produces the soluble ICAM-1 (sICAM-1). [3, 6] The function of membrane-bound and soluble ICAM-1 is described in the following section.

## ICAM-1 function

### Membrane-bound ICAM-1 (mICAM-1)

The function of ICAM-1 is largely studied on full-length mICAM-1. In addition to LFA-1, mICAM-1 binds to macrophage-1 antigen (Mac-1) and aXb2 (p150,90). LFA-1, Mac-1 and aXb2 belong to the adhesion molecule family known as b2 integrins. These adhesion molecules are expressed only in leukocytes. The importance of these b2 integrins is well appreciated in a genetic disease called Leukocyte Adhesion Deficiency Type I (LAD-1); LAD-1 is caused by the loss of expression or function of b2 integrins and is characterized by impaired neutrophil recruitment and recurrent soft tissue infections, leading into death sometimes. [7, 8] As seen in a sepsis model using full-length mICAM-1 deficient mice, the lack of mI-CAM-1 expression impaired neutrophil recruitment to the liver, suggesting that mICAM-1 also plays a critical role in neutrophil recruitment. [9] The interaction of mICAM-1 with these molecules clearly affects the function of leukocytes.

The binding of b2 integrins to mICAM-1 depends on dynamic, structural changes of the integrins from the resting conformation to the active conformation in response to activation signals. The b2 integrins bind to mICAM-1 only in their active conformation. [10, 11] Pro-inflammatory cytokines and chemoattractants trigger intracellular signaling by interacting with leukocytes, and activate b2 integrins (inside-out signaling). In the early perioperative period, strong pro-inflammatory signals allow for appropriate activation of β2 integrins, which is necessary for an adequate immune response.

LFA-1 and Mac-1 are studied most among b2 integrins. LFA-1 is expressed on all leukocytes, while Mac-1 is predominantly expressed on neutrophils, monocytes, and macrophages. The expression level of aXb2 in neutrophils is low, but monocytes and macrophages highly express aXb2. [12] Because neutrophils are the first-line defense cells in innate immunity and most relevant to perioperative immunity, we will focus on LFA-1 and Mac-1, the major b2 integrins that are expressed on neutrophils. LFA-1 and Mac-1 bind to mICAM-1 at different domains: LFA-1 binds to D1 of mICAM-1, whereas Mac-1 binds to D3 (Figure 2). Theoretically, it is possible for LFA-1 and Mac-1 to bind to mICAM-1 simultaneously because the ICAM-1 domains to which each adhesion molecule binds are sufficiently far apart. However, this has not been investigated in an experimental setting. In addition, there is a difference in binding affinity of these two integrins to mICAM-1. The binding affinity between mICAM-1 and Mac-1 is weaker than that between mICAM-1 and LFA-1.[13] Full-length mICAM-1 exists primarily as a dimer on the cell surface. The binding of LFA-1 to mI-CAM-1 becomes two-fold stronger when mICAM-1 is present as a dimer, presumably because dimerization allows mICAM-1 to properly orient on the cell surface.[14] However, the comparison between the binding of Mac-1 to the ICAM-1 dimer and that of the monomer has not yet been reported. It is unclear if dimerization is responsible for the difference in binding affinity between LFA-1 and Mac-1. Glycosylation also plays a role in function by affecting the binding ability of Mac-1 to mICAM-1. [15] mICAM-1 likely undergoes different types of glycosylation in various tissues. It is likely that Mac-1 binds to mICAM-1 with different affinities in different tissues.

Although both LFA-1 and Mac-1 bind to mICAM-1, they may have different functional roles in leukocyte recruitment. Some leukocytes, such as the majority of T cells and B cells, express only LFA-1, while neutrophils, for example, express both. This difference in tissue distribution of b2 integrin expression accounts for the functional difference of these integrins. Both LFA-1 and Mac-1 are highly expressed in neutrophils. During recruitment, neutrophils undergo various steps: capture, rolling, slow rolling, arrest (adhesion) and transmigration. On endothelium in which inflammation has been stimulated by TNF-α, the slow rolling of neutrophils was predominantly dependent on Mac-1: mICAM-1 interaction. [16] LFA-1 plays a significant role in neutrophil adhesion, [17, 18] but Dunne et al. suggested that ICAM-1 is not a ligand for LFA-1 in neutrophil adhesion to TNF-α-stimulated endothelium. [16] Foy et al. also studied the role of mICAM-1 in neutrophil adhesion to the endothelium with or without TNF-α stimulation in cremaster muscle.[19] They found that mICAM-1 is a ligand for neutrophil adhesion to the unstimulated endothelium, but that it is not a ligand for neutrophil adhesion to the TNF-α-stimulated endothelium despite high ICAM-1 expression. It is unclear if these results are applicable to neutrophil recruitment in general, or if they only apply to some tissues including cremaster muscle, because the *in vitro* study using a flow chamber assay by Green et al. showed that LFA-1: ICAM-1 interaction is required for neutrophil adhesion to the IL-1β stimulated endothelium.[20] Of note, the endothelial cells of skeletal muscle beds have little in common with endothelial cells of the lung, liver or kidney, supporting the possibility that mICAM-1 may play a different role in neutrophil adhesion, depending on the tissues in which it is expressed. [19] A deficiency of mICAM-1 hinders recruitment, but increased expression of ICAM-1 during inflammation is not associated with enhanced neutrophil recruitment. The *in vitro* study by Yang et al. did not find any change in the degree of neutrophil transmigration in TNF-α-activated endothelium.[21] Interestingly, however, their study demonstrated that the mode of transmigration changes with higher ICAM-1 expression. Generally, neutrophils can transmigrate between endothelial cells (paracellular migration) and through the endothelial cells (transcellular migration). Increased ICAM-1 expression facilitated more transcellular migration. Overall, ICAM-1 plays a significant role in neutrophil recruitment, but whether or not it plays a different role in different tissues needs further clarification.

### ICAM-1 Isoforms

Several studies have shown that ICAM-1 isoforms are functional *in vivo.* [22] These studies were performed using ICAM-1 transgenic mice that were intended to be knockout mice by deleting one exon in their ICAM-1 gene. However, instead of serving as ICAM-1 knockout mice, these exon-deletion transgenic mice expressed ICAM-1 isoforms. Exon 4 deletion mice express D1D4D5, D1D2D5 and D1D5, while exon 5 deletion mice express D1D2D3D5, D1D2D5 and D1D5. In the shock model using intraperitoneal injection of lipopolysaccharide (LPS), Robledo et al. found that wild type mice had a 50% survival rate, while exon 5-deletion mice showed a 23% survival rate, and 100% of exon 4-deletion mice survived. [23] Among exon 5-deletion mice, a large amount of neutrophil recruitment was observed in the liver, while neutrophil recruitment to the liver was minimal among exon 4-deletion mice. The distribution of these ICAM-1 isoforms among different tissues and their functions in relation to LFA-1 and Mac-1 needs further investigation, but this study suggests that ICAM-1 isoforms have an important role in neutrophil recruitment. Although full-length mICAM-1 expression is inducible, it is not known whether expression of ICAM-1 isoforms is inducible.

### Soluble ICAM-1 (sICAM-1)

sICAM-1 can shed from the cell surface via a disintegrin and metalloproteinase (ADAM)-17 and neutrophil elastase (NE). sICAM-1 can exist as a monomer or as a dimer.[6] The binding strength of sICAM-1 to LFA-1 significantly depends on its dimerization. Dimer sICAM-1 showed 10–100 times as high binding affinity to LFA-1 as monomer sICAM-1.[14] The binding of sICAM-1 to Mac-1 has not yet been reported, but our experiments did not find significant binding between them. Various investigators reported that sICAM-1 competitively inhibited LFA-1: ICAM-1 interaction. [24–26] Rieckmann et al. demonstrated that sICAM-1 at 150 – 200 ng/mL (~3–4 nM) inhibited the binding of peripheral blood mono-nuclear cells (PBMCs) to cerebral endothelial cells [24], but it is unclear if the sICAM-1 was a dimer or a monomer. In addition, they showed that serum of patients suffering from multiple sclerosis inhibited the binding as well, suggesting that sICAM-1 in the human body could act as an inhibitor. The subsequent study by Meyer et al. examined the effect of sICAM-1 (recombinant sICAM_453_) on the adhesion of SKW cells (LFA-1 expressing cells) to ICAM-1 and RAJI cell aggregation, and found that sICAM_453_inhibited LFA-1: ICAM-1 interaction with the IC_50_ of 20–40 μM.[25] However, the authors recognized an accidental mutation in the recombinant sICAM-1, and also this sICAM-1 may have been expressed as a monomer. By contrast, the study by Jun et al. showed of 800 nM.[26] Overall, that dimer sICAM-1 had IC_50_ a wide range of IC_50_s have been reported. This could be due to the abundance of dimerized sICAM-1 in the prepared solution, as well as other experimental conditions. Because the concentration of circulating sICAM-1 in human subjects is around 500 ng/mL (~10 nM) [27] and serum from patients with multiple sclerosis inhibited LFA-1: ICAM-1 interaction, it is likely that sICAM-1 acts as an inhibitor *in vivo*. Knowing that sICAM-1 binds to LFA-1, it needs to be determined whether sICAM-1 can inhibit the interaction of LFA-1 with all ligands.

## Perioperative implications of ICAM-1

### The role of ICAM-1 in perioperative disease models

Ischemia-reperfusion is observed in various surgeries as a part of surgical procedures or after relief of vascular obstruction. And an extreme neutrophil recruitment as a result contributes to a tissue injury. The role of ICAM-1 in ischemia-reperfusion injury has been studied in various organs in animal models. A 45-minute of liver ischemia followed by reperfusion induced liver injury in rats, which was attenuated by ICAM-1 neutralizing antibody. (29) Similarly a 30-minute of myocardial ischemia by ligation of a coronary artery followed by reperfusion caused a significant myocardial necrosis in wild-type mice, while ICAM-1 knockout mice had less necrotic lesion.(30) The study by Kusterer et al. demonstrated that neutrophil adhesion following ischemia-reperfusion was attenuated by the administration of recombinant soluble ICAM-1 protein (31). Although not conclusive, Shimada et al. described the increased ICAM-1 expression in liver after partial hepatectomy than in liver prior to resection in patients, and suggested that this might be from ischemia-reperfusion injury as a result of Pringle maneuver for hepatectomy.(32) Of note, serum soluble ICAM-1 level was lower after resection than before resection, suggestion a possibility to attenuate liver injury with ICAM-1 as a target.

### Future research directions of ICAM-1 in the perioperative setting

mICAM-1 undoubtedly plays a significant role by assisting leukocyte recruitment in the perioperative setting, but the role of mICAM-1 during various circumstances has yet to be investigated. Ischemia-reperfusion injury is such an example. The role of mI-CAM-1 in leukocyte recruitment to the un-inflamed endothelium versus the inflamed endothelium in different tissues must be defined. Additionally, the role of mICAM-1 upregulation on the inflamed endothelium in various tissues has not been determined, although mICAM-1 is not a primary ligand in neutrophil adhesion to the inflamed endothelium in cremaster muscle. Because various pro-inflammatory cytokines are produced in the perioperative period, the endothelium in patients becomes inflamed. Some surgical procedures cause minimal stress responses, while procedures such as cardiac surgery in cardiopulmonary bypass can trigger an exaggerated pro-inflammatory response. This can cause a robust mICAM-1 upregulation on the endothelium. Although adequate neutrophil recruitment is beneficial for tissue health, an exaggerated accumulation of neutrophils is harmful. If mICAM-1 facilitates neutrophil recruitment to the significantly inflamed endothelium in certain tissues, this can lead to tissue destruction.

In order to study the role of mICAM-1 in these processes, the level of mICAM-1 expression must be determined. Because it is unrealistic to measure the levels of mICAM-1 levels on the endothelium from a clinical standpoint, an alternative method is warranted. sICAM-1 is easily measured by obtaining serum or plasma. Zonneveld et al. proposed a model of changes to mICAM-1 and sICAM-1 during progression of inflammatory responses [28]. Inflammation increases cell-surface expression of mICAM-1. Subsequently the level of mICAM-1 decreases during the resolution of inflammation as sICAM-1 increases. If aberrant shedding occurs along with sustained inflammation, the sICAM-1 level may not increase significantly. The level of sICAM-1 depends on the expression level of mICAM-1 and cleavage by sheddases such as ADAM17 and NE. This may explain why the level of sICAM-1 does not always correlate with disease outcomes in various disease processes, including sepsis.[28] In order to predict mICAM-1 expression levels, it is critical to understand expression levels of sICAM1, ADAM17, and NE.

Once these questions are answered, we need to understand if we can modulate mICAM-1 function. The administration of sICAM-1 may modulate mICAM-1 functions related to LFA-1. Less invasive surgical procedures and pharmacological interventions are likely candidates for modulating the function of mICAM-1. Although ICAM-1 has been studied for about 30 years since its discovery, there are still many questions to investigate. The elucidation of these questions, however, will allow us to understand patients’ immune function more thoroughly and therefore opens a pathway to modulate those functions for patient benefit.
